# Early Prediction of Outcome Using Lactate Dehydrogenase and Amplitude‐Integrated EEG in Transported Newborn Infants with Hypoxic–Ischaemic Encephalopathy

**DOI:** 10.1111/apa.70556

**Published:** 2026-04-30

**Authors:** Hang T. T. Tran, Ha T. Le, Dien M. Tran, Tobias Alfvén, Linus Olson, Lena Hellstrom‐Westas

**Affiliations:** ^1^ Department of Global Public Health Karolinska Institutet Stockholm Sweden; ^2^ Neonatal Care Center Vietnam National Children's Hospital Hanoi Vietnam; ^3^ Vietnam National Children's Hospital Hanoi Vietnam; ^4^ Sach's Children's Hospital Stockholm Sweden; ^5^ Department of Women's and Children's Health Karolinska Institutet Stockholm Sweden; ^6^ Department of Women's and Children's Health Uppsala University Uppsala Sweden

**Keywords:** biomarker, birth asphyxia, hypoxic–ischaemic encephalopathy, lactate dehydrogenase, therapeutic hypothermia

## Abstract

**Aim:**

To investigate whether early lactate dehydrogenase (LDH) and amplitude‐integrated electroencephalography (aEEG) improve the prediction of outcome in outborn infants transported for therapeutic hypothermia.

**Method:**

Secondary analysis of a randomised controlled trial (2016–2019) including 113 asphyxiated newborn infants randomised to transport with a phase‐change material mattress or standard care. The analysis included 81 infants with available aEEG, of whom 50 had LDH measured on admission. Outcome at 18 months was categorised as good, defined as normal development or mild delay, or poor, defined as moderate or severe impairment or death.

**Result:**

Mean (SD) rectal temperature on admission was lower in the PCM group (34.6 [1.1]°C vs. 35.2 [1.1]°C, *p* = 0.027), but the proportion within target temperature did not differ (39.5% vs. 25.6%, *p* = 0.235). Clinical characteristics, LDH, aEEG and outcomes were similar between groups. Poor outcome was predicted by LDH > 1000 U/L (89% sensitivity and 61% specificity), severely depressed aEEG within 12 h (87% sensitivity and 47% specificity) and the combination of both (91% sensitivity, 62% specificity and accuracy 81%).

**Conclusion:**

Combined LDH and aEEG provide a strong early prediction of outcome in encephalopathic infants.

## Summary

- Early clinical evaluation of transported infants with HIE may be challenging, not least when information from, for example, early blood gas analyses, may be lacking.
- Results from a few studies have previously indicated that high blood levels of LDH may distinguish asphyxiated infants at high risk for poor outcomes.
- The presented data showed that in asphyxiated infants transported for hypothermia treatment, LDH on arrival, together with early aEEG, were highly predictive of outcomes at 18 months.

**Abbreviations**
aEEG: amplitude‐integrated electroencephalography
ASQ: Ages and Stages Questionnaires
HIE: hypoxic–ischaemic encephalopathy
HINE: Hammersmith Infant Neurological Examination
LDH: lactate dehydrogenase
LMIC: low‐ and middle‐income countries
MRI: magnetic resonance imaging
PCM: phase‐change material
TH: therapeutic hypothermia
VNCH: Vietnam National Children's Hospital

## 1 Background

Hypoxic–ischaemic encephalopathy (HIE) affects approximately 3–5 per 1000 live‐born term births, among which 10%–30% do not survive, and 25%–30% of survivors face long‐term neurodevelopmental problems [1]. Studies in high‐income countries have demonstrated the efficacy and safety of therapeutic hypothermia (TH) as treatment for moderate–severe HIE [2, 3]. In contrast, the potential benefits of TH in low‐ and middle‐income countries (LMIC) are currently discussed [4].

TH was introduced at the Vietnam National Children's Hospital (VNCH) in 2014, and coupled with a training programme for resuscitation at the referring local hospitals [5]. The first observational study was followed by a randomised study evaluating phase‐change material (PCM) during inter‐hospital transport of 113 newborns eligible for TH at VNCH. The results demonstrated that infants transported with PCM had, on average, 0.6°C lower body temperature on arrival without overcooling [6].

Early accurate evaluation of infants with encephalopathy is crucial since the potential benefits and risks of TH in newborns with conditions other than moderate or severe encephalopathy have not been evaluated in randomised investigations [7]. The amplitude‐integrated electroencephalogram (aEEG) has demonstrated a significant capacity to accurately predict outcome following birth asphyxia [8]. Among the biomarkers studied, lactate dehydrogenase (LDH) has been shown to predict HIE with high sensitivity and specificity at defined cut‐off levels [9, 10].

We hypothesised that interhospital transport using PCM would not significantly alter early LDH levels or aEEG background patterns compared with standard passive cooling, given the relatively small differences in achieved body temperature. We further hypothesised that elevated LDH on admission and severely depressed early aEEG background activity, individually and in combination, would be associated with adverse neurodevelopmental outcomes at 18 months of age.

The objective was to evaluate whether interhospital transport using phase‐change material was associated with differences in lactate dehydrogenase levels or aEEG patterns and whether these biomarkers predicted outcome.

## 2 Method

This study was a secondary analysis of a randomised controlled trial conducted between 2016 and 2019, evaluating transport of asphyxiated newborn infants using phase‐change material compared with standard care [6].

Eligible infants participated in a randomised controlled trial (RCT), evaluating inter‐hospital transport of asphyxiated infants with PCM (cooling mattress), compared to standard management (passive cooling), from 2016 to 2019 [6]. The PCM mattress was purpose‐built (*Medical Cooling Sweden AB; by TST AB, Kinna, Sweden*, melting point 32°C) and used in a randomised fashion during transport from referral hospitals to VNCH. After arrival at VNCH, the PCM mattress was used for all cooled infants receiving TH. Clinical criteria for HIE and eligibility for TH were adapted from the TOBY trial, and the severity of HIE was assessed on admission according to the modified Sarnat stages as mild, moderate or severe [2, 11]. The study design included continuous aEEG monitoring in all infants during TH, but due to technical problems and lack of equipment, full recordings failed in many children. As a result, the current study population consisted of 81 infants for whom aEEG recordings had been performed continuously for at least 72 h of TH or until the infant died. LDH was randomly measured on arrival in 50 of these infants. Blood cultures were sampled after arrival to rule out sepsis. Brain MRI was performed at 7–14 days. Surviving children had follow‐up at 6 and 18 months, and outcomes were classified as normal, mild delay, cerebral palsy/epilepsy and death, as previously reported [12].

### 2.1 Ethics Statement

The original study was approved by the ethical committee of the National Hospital of Paediatrics (now Vietnam National Children's Hospital), and written informed consent was obtained from parents.

### 2.2 Study Population and Flow of Participants

A total of 113 infants were enrolled in the original randomised trial. Of these, 81 infants had continuous aEEG recordings of sufficient quality and duration to allow analysis and were included in the present study. LDH on admission was available in 50 of these infants.

**FIGURE 1:**
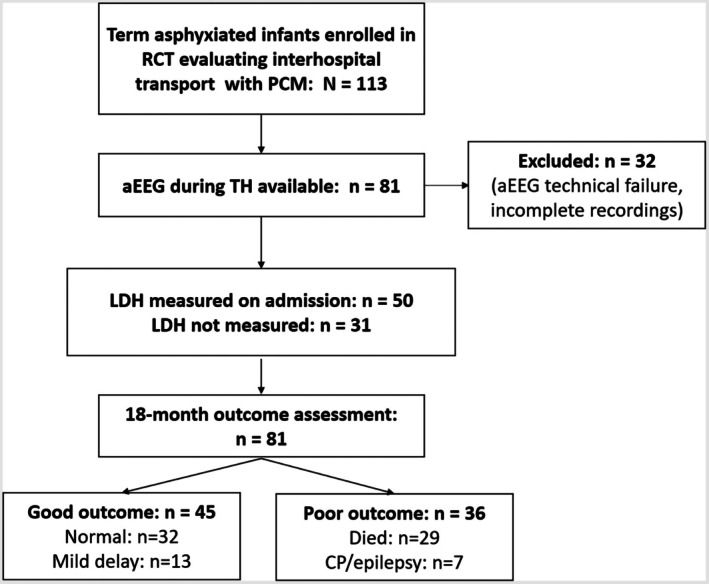
Flow of participants from enrolment in the randomised transport trial to final analysis.

The flow of participants from enrolment to final analysis is illustrated in Figure 1.

### 2.3 Clinical Management

Target rectal temperature 33.5°C–34.5°C was maintained and monitored every hour for 72 h, followed by slow rewarming (temperature increase 0.5°C/h).

During TH, systemic support was provided, including mechanical ventilation, cardiovascular support and anti‐convulsive medications as required (see Table 2). The infants were ventilated throughout the 72 h of cooling and the rewarming phase and received a continuous infusion of morphine (10 mcg/kg/h). Seizures, when detected clinically or on aEEG, were treated with first‐line phenobarbitone 20 mg/kg/dose, then maintenance of 10 mg/kg/dose, and when required also with midazolam and/or levetiracetam.

### 2.4 Amplitude‐Integrated Electroencephalography

Continuous aEEG (Olympic CFM 6000 Monitor; Olympic Medical, Seattle, WA, and USA) was recorded until the end of the rewarming period, according to the protocol. The aEEG recordings were digitally stored, and a summary of the aEEG pattern was printed and stored in each patient's medical record for reference.

The aEEG traces were assessed (HT and LHW), blinded to the patients' clinical condition. The background pattern was determined from bilateral (left and right frontal‐central leads) and cross‐cerebral central leads, using a slightly modified scoring system based on a previously published classification [13]. aEEG background scores were scored for each 6‐ and 12‐h period after birth up to 72+ hours. The scores were based on the dominating background patterns within each time‐period, that is, continuous normal voltage (CNV) = score 5; discontinuous normal voltage (DNV) = score 4; dense burst‐suppression+ (≥ 100 bursts/h) = score 3; sparse burst suppression‐ (< 100 bursts/h) = score 2; low voltage (LV) and flat/isoelectric (FT) = score one. If more than one pattern appeared in a 6‐h period, then an average score of the dominating patterns was given, that is, FT/BS‐ = score 1.5, BS‐/BS+ = score 2.5, BS+/DC = score 3.5, DC/CNV = score 4.5. For the 12‐h epochs, mean scores for the two included 6‐h periods were created. For each period, seizures (single seizures or status epilepticus, verified by the simultaneous raw‐EEG trace). The raw EEG was assessed for each suspected seizure observed in the aEEG trend. Furthermore, for each 10‐min record of the aEEG, 25 s of raw EEG were inspected throughout the recordings to exclude prolonged seizures or artefacts that were not obvious in the aEEG trend. A single seizure was defined by an increase, maximum and decrease in amplitude and frequency of rhythmic activity in the raw EEG with a duration of at least 10 s [14]. Status epilepticus was defined as continuous or repetitive seizures with a duration of at least 30 min. Sleep–wake cycling (SWC) (absent, immature and mature) was continuously evaluated but not included in the scoring.

### 2.5 Follow‐Up and Outcome Assessment

Survivors underwent neurodevelopmental follow‐up at 6 and 18 months of age by a multidisciplinary team including a neonatologist and a rehabilitation physician. Assessment tools included the Ages and Stages Questionnaire (ASQ) and the Hammersmith Infant Neurological Examination (HINE).

Outcomes were categorised as: normal development, mild developmental delay, cerebral palsy and/or epilepsy or death. For prognostic analyses, outcomes were dichotomised into good outcomes (normal or mild delay) and poor outcomes (death or survival with cerebral palsy and/or epilepsy).

Standardised cognitive testing using the Bayley Scales of Infant and Toddler Development was not systematically available during the study period due to resource and training limitations.

### 2.6 Statistical Analysis

Data were collected in an Excel database and analysed using the IBM SPSS Statistics 28.0 and MedCalc Software Ltd. Diagnostic test evaluation calculator (https://www.medcalc.org/calc/diagnostic_test.php, Version 22.009; accessed August 22, 2023). Clinical characteristics and outcomes were presented with absolute numbers (percentages) for categorical variables and means (SD) or medians (IQR) for continuous variables. Group differences in outcome were analysed by Chi‐square tests, Fisher's exact tests, Mann–Whitney U tests and ANOVA. Sensitivity, specificity, positive predictive value (PPV), negative predictive value (NPV) and accuracy (proportion of correctly predicted infants at a certain cut‐off level) were reported for adverse outcome. Two‐sided tests were used, and a *p* value below 0.05 was considered statistically significant.

Repeated‐measures or mixed‐effects models were considered; however, these approaches were not feasible due to substantial missing data across time points, variable duration of aEEG recordings and early deaths during therapeutic hypothermia. To minimise bias and preserve sample size, analyses were therefore performed at predefined clinically relevant time intervals.

## 3 Results

The study population is presented in Tables 1 and 2. Gestational ages ranged from 36 to 41 weeks. Apgar scores were available in 34/81 (42%) infants and lacking in 47/81 (58%). Thirty‐eight of 81 infants (47%) were transported using a phase‐change material mattress, while 43/81 (53%) received standard care. Mean temperature on admission was lower in the PCM group (34.6°C [SD 1.1] vs. 35.2°C [SD 1.1], *p* = 0.027). Fourteen infants in each randomisation group had clinically suspected seizures before or at admission (*p* = 0.299). Thirty infants received phenobarbitone and/or midazolam for clinically suspected seizures prior to admission at VNCH. Six of the 27 nonsurviving infants died before completing TH due to pneumothorax (*n* = 3), pulmonary hypertension (*n* = 1), sepsis (*n* = 1) and necrotising enterocolitis (*n* = 1).

**TABLE 1:** Secondary analysis of 81 infants with available aEEG recordings, out of 113 asphyxiated newborn infants randomised to inter‐hospital transport with a PCM mattress or passive cooling (standard) for hypothermia treatment at Vietnam National Children's Hospital (VNCH).

	PCM group (*N* = 38)	Control group (*N* = 43)	*p*
Gestational age, w	39.0 (1.1)	39.0 (1.2)	0.937
Birth weight, *g*	3095 (391)	3059 (411)	0.690
Apgar score, 10 min	4.9 (1.6), *n* = 17	4.8 (2.0), *n* = 17	0.398
Clinical seizures during transport, *n*	14 (26%)	11 (26%)	0.278
Temperature on admission, °C	34.6 (1.1)	35.2 (1.1)	0.027
Target therapeutic temperature (33.5°C–34.5°C) on admission	15 (39.5%)	11 (25.6%)	0.235
Postnatal age (h) at start of TH after arrival at VNCH	3.9 (1.5)	4.3 (1.5)	0.238
LDH on admission, median (IQR) U/L	1385 (906–4298), *n* = 28	1279 (851–1832), *n* = 22	0.190
LDH > 1000 U/L, *n* (%)	18 (64%)	15 (68%)	0.773
HIE III, *n*	29 (76.3%)	26 (60.5%)	0.147
Mechanical ventilation, *n*	42 (97.7%)	37 (97.4%)	0.929
Inotropes, *n*	27 (71.1%)	20 (46.5%)	0.026
Kidney/liver failure, *n*	20 (52.6%)	17 (39.5%)	0.238
Normal brain MRI, *n*	14 (42.4%), *n* = 33	18 (52.9%), *n* = 34	0.389
Neonatal death, *n*	11 (28.9%)	16 (37.2%)	0.431

*Note:* Values are numbers, percentages, mean (standard deviation), median (IQR), as appropriate.

**TABLE 2:** Development of mean (standard deviation) aEEG background scores during TH in infants transported with the PCM group (*n* = 38) compared to infants with passive cooling during transport (*n* = 43).

	PCM group (*N* = 38)	Control group (*N* = 43)	*p*
aEEG score* 0–12 h	2.4 (1.4), *n* = 37	2.2 (1.1), *n* = 42	0.535
aEEG score* 12–24 h	2.5 (1.5), *n* = 37	2.2 (1.3), *n* = 42	0.305
aEEG score* 24–36 h	2.5 (1.6), *n* = 36	2.3 (1.4), *n* = 41	0.563
aEEG score* 36–48 h	2.9 (1.6), *n* = 45	2.6 (1.5), *n* = 37	0.556
aEEG score* 48–60 h	3.0 (1.6), *n* = 29	2.7 (1.9), *n* = 28	0.893
aEEG score* 60–72 h	3.0 (1.4), *n* = 29	2.9 (1.7), *n* = 28	0.832
aEEG status epilepticus, *n*	15 (39.5%)	15 (34.9%)	0.669
Normal/mild outcome, *n*	18 (47.4%)	25 (58.1%)	0.332

* aEEG background scores were given for each 6‐h period (score: 1 = Flat; 2 = BS‐; 3 = BS+; 4 = DNV; 5 = CNV). In the 12‐h periods presented above, the mean scores for two 6‐h epochs are presented.

LDH on admission was measured in 50/81 (62%) infants, including 28/38 (74%) in the PCM group and 22/43 (51%) in the control group (*p* = 0.056). LDH values above 1000 U/L were observed in 33/50 (66%) infants, including 24/27 (89%) with poor outcome and 9/23 (39%) with good outcome (*p* < 0.001).

**FIGURE 2:**
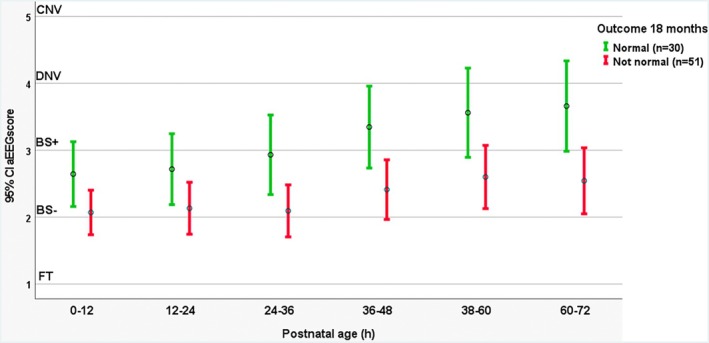
Mean (95% CI) aEEG scores during the first 72 h in relation to outcome at 18 months, in infants with normal development (green) compared to infants with mild/moderate/severe (including death) outcomes (red). The differences between the groups did not reach statistical significance.

The initial aEEG background activity was depressed in a majority of the infants, with average scores of around two corresponding to burst suppression or mixed flat trace and burst suppression patterns up to 36 h postnatal age. The median aEEG scores for each 12‐h period in the four outcome groups are presented in Figure 2. Overall, 35/81 (43%) infants developed a continuous normal voltage pattern within 72 h, at a median age of 16 h (IQR 5–35). Immature SWC developed within 72 h in 28/81 (35%) infants, while it developed later, between 73 and 138 h, in 14/81 (17%) infants. No SWC was observed in 37/81 (46%) infants. The postnatal age when CNV and SWC developed during the first 72 h of TH did not differ between the two randomisation groups.

Mean aEEG background scores during the first 72 h of TH did not differ significantly between infants transported with PCM and those receiving standard passive cooling at any time interval.

During TH, seizures were clinically recognised in 45 infants, and subsequently treated with anti‐seizure medications including phenobarbitone, midazolam and/or levetiracetam. Overall, the aEEG (verified by the raw EEG) showed seizures in 61 infants, including 30 infants (37%) with status epilepticus.

MRI scans were done at 7–10 days of age in 67/81 (83%) infants. Ten of the 14 infants without an MRI died in the neonatal period. The MRIs were clinically assessed by trained radiologists, and the dominating abnormalities were classified either as basal ganglia injury/abnormal signal in the posterior limb of the internal capsule (PLIC) (*n* = 15), or white matter injury/intraventricular haemorrhage/brain oedema (*n* = 20), or no major abnormalities (*n* = 32).

### 3.1 Neurodevelopmental Outcome at 18 Months

At 18 months, outcome was classified as good in 45/81 (56%) infants, including 32/81 (40%) with normal development and 13/81 (16%) with mild developmental delay. Poor outcome was observed in 36/81 (44%) infants, including 29 deaths (36%) and seven survivors (9%) with cerebral palsy and/or epilepsy.

### 3.2 Prediction of Outcome

As no significant differences were observed between the PCM and control groups with respect to LDH levels, aEEG background scores or neurodevelopmental outcomes, the two groups were pooled for analyses evaluating the prognostic performance of LDH and aEEG. This approach was chosen to maximise statistical power and to focus on biomarker prognostication independent of transport modality.

The sensitivity and specificity of an LDH value above 1000 U/L for predicting poor outcome were 89% and 61% respectively (Table 3). The combined predictive values for clinical seizures on admission and high LDH showed a high specificity (96%) and PPV (88%) for predicting poor outcome [15].

**TABLE 3:** Predictors of poor outcomes (death or survival with cerebral palsy or epilepsy) in 81 asphyxiated newborn infants undergoing TH at the Vietnam National Children's Hospital in Hanoi.

	Sensitivity (%)	Specificity (%)	Accuracy (%)	PPV (%)	NPV (%)	*p*
Admission (*n* = 81/50)						
Clinical seizures before/on admission, yes (*n* = 25)	39	**77**	59	60	59	0.115
LDH > 1000 U/L (*n* = 35)	**89**	61	**76**	73	**82**	**< 0.001**
Clinical seizures and LDH > 1000 (*n* = 25)	26	**96**	58	**88**	52	**0.038**
aEEG 0–12 h (*n* = 81)						
aEEG BG score < 3	**87**	47	65	59	**80**	**0.001**
aEEG BG score < 3 and LDH > 1000 (*n* = 35)	**91**	62	**81**	**81**	**80**	**0.007**
aEEG 12–24 h (*n* = 79)						
aEEG BG score < 3	**78**	40	57	52	68	0.094
aEEG BG score < 3 and LDH > 1000 (*n* = 33)	**90**	62	**79**	**79**	**80**	**< 0.001**
aEEG 24–36 h (*n* = 77)						
aEEG BG score < 3	**80**	48	62	56	74	**0.011**
aEEG BG score < 3 and LDH > 1000 (*n* = 32)	**90**	50	**75**	**75**	**75**	**< 0.001**
aEEG 36–48 h (*n* = 71)						
aEEG BG score < 3	70	61	65	61	70	**0.011**
aEEG 48–60 h (*n* = 65)						
aEEG BG score < 3	41	64	54	48	58	0.070
aEEG 60–72 h (*n* = 57)						
aEEG BG score < 3	74	47	60	56	67	0.105
Other predictors of poor outcome (*n* = 81)						
Flat aEEG anytime duration first 24 h	**74**	60	67	62	72	**0.006**
Flat aEEG first 24 h + LDH > 1000 (*n* = 48)	**80**	74	**77**	**77**	**77**	**< 0.001**
HIE grade 3 (*n* = 50)	68	33	49	47	54	0.925
Status epilepticus (*n* = 30)	47	72	60	60	61	0.070
Organ failure (*n* = 37)	66	72	69	68	70	**< 0.001**
Inotropes (*n* = 47)	**76**	58	67	62	74	**0.002**

*Note:* LDH was measured in 50 infants upon arrival. Chi square statistics for differences between the two outcome groups. Numbers in bold highlight predictive values of 75% and above, or *p* < 0.05 for differences between the two outcome groups.

A severely depressed aEEG pattern (i.e., score below 2) in the first 24 h had 75% sensitivity and 40% specificity for predicting poor outcome. The predictive performance of combined LDH and aEEG is summarised in Table 4. However, when combined with a high LDH (> 1000 U/L), the sensitivity and specificity increased to 91% and 62% respectively.

**TABLE 4:** Predictors of ‘good’, that is, normal‐mild, outcomes at 18 months in relation to mode of transport (PCM), MRI and presence of discontinuous/continuous aEEG activity (scores 4–5) at different time‐points, and presence of sleep–wake cycling (SWC) respectively.

	Sensitivity (%)	Specificity (%)	Accuracy (%)	PPV (%)	NPV (%)	*p*
PCM (transport) (*n* = 38/81)	42	47	44	47	42	0.332
Normal MRI (*n* = 67)	**88**	63	**78**	**79**	**76**	**< 0.001**
aEEG BG score 4–5 at 0–24 h	21	**87**	52	64	49	0.356
aEEG BG score 4–5 at 24–48 h	42	**81**	59	73	53	**0.044**
aEEG BG score 4–5 at 48–72 h	50	**77**	66	53	66	**0.049**
SWC, any 0–72 h	33	**79**	54	64	51	0.245

*Note:* Numbers in bold aim at highlighting the results and indicate values at 75% and over, and statistically significant differences between outcome (good vs. poor) and variable (present/not present) on Chi square analysis.

## 4 Discussion

This is a secondary analysis of an RCT investigating the transport of asphyxiated newborn infants with a cooling mattress made of PCM. Here, we demonstrated that LDH and aEEG early after arrival were highly predictive of outcome in encephalopathic infants and contributed important information on the infants' condition. The primary outcome showed that body temperature upon arrival at VNCH was lower in the PCM group than in controls, but no infant was overcooled, and there were no differences in outcomes [6].

Inclusion criteria in the RCT were adapted from the TOBY trial, but it was not possible to fulfil all criteria. Blood gas measurements were not available in most referring hospitals, and there was traditionally some reluctance to give poor Apgar scores, which is reflected in the large proportion of infants lacking Apgar scores. The decision to transport the infant was based on telephone consultation with the receiving neonatologist, including information on delivery, need for resuscitation and clinical signs of encephalopathy.

In this secondary analysis of the randomised trial, no differences were observed between the transport groups in aEEG background patterns during the first 72 h of therapeutic hypothermia. Rather than reflecting an absence of effect, this finding is likely explained by the relatively small difference in admission temperature between the groups. Previous studies have shown that adverse outcomes are primarily associated with hyperthermia, whereas modest temperature differences within the hypothermic range are less likely to influence neuroprotection [4, 16]. The predictive value of aEEG in this cohort was lower than reported in previous studies and meta‐analyses [16, 17], which may be explained by clinical factors such as the frequent use of anti‐seizure medications during transport and the inclusion of infants with heterogeneous clinical conditions. These findings highlight the importance of considering clinical context when interpreting early neurophysiological markers in low‐resource settings.

A few observational studies have indicated that LDH levels below 1049 U/L and also below 2085 U/L, respectively, are predictive of better outcomes in asphyxiated infants [15, 18, 19]. In the present study, LDH levels on arrival varied widely but did not differ between the two randomisation groups. Previous investigations have demonstrated that LDH activity remains unaffected by the narrow temperature ranges employed in TH [20], in contrast to other prognostic biomarkers that are influenced by TH treatment [21]. Nevertheless, clinical seizures during transport, or an initially severely depressed aEEG, respectively, combined with information from an LDH measurement on arrival, were strongly predictive of long‐term outcome in this cohort with limited information on the early postnatal course, such as Apgar scores, umbilical cord or early blood gases.

Similar results to the present study, on the value of LDH for predicting neurodevelopmental prognosis in HIE, have been reported by Yum et.al. [18].

Evaluation of neurologic symptoms and aEEG together shortly after birth improves identification of high‐risk infants who could benefit from TH [20]. In the present study, the predictive value of aEEG was lower than in previous studies. This could be due to several circumstances: a relatively large proportion of infants received phenobarbital for suspected seizures during transport, which could depress the aEEG. Another explanation could be that the study population also included infants with other conditions, as discussed above. Although all infants required resuscitation and were considered asphyxiated at birth, more than half of the infants did not have Apgar scores assigned, and no infant had blood gas analysis before transport.

A clinically important finding in the present study was that the combined information from LDH and aEEG increased the early predictive accuracy by 10%–20%. High LDH on admission (> 1000 U/L) together with a severely depressed aEEG had a very high sensitivity (90%) and specificity (82%) for predicting adverse outcome, with levels comparable to a systematic review by Del Rio et al. [17].

### 4.1 Strengths and Limitations

In spite of the novel findings in this study, demonstrating the high performance of LDH and aEEG combined in infants with suspected hypoxic–ischaemic encephalopathy, there are several limitations. Major limitations are the lack of early blood gases and Apgar scores, and also that we did not succeed in measuring LDH and recording aEEG in all admitted infants. Although structured neurological examination, ASQ and the HINE were used by trained assessors, longer‐term follow‐up with validated cognitive assessments is required to fully characterise neurodevelopmental outcomes.

## 5 Conclusions

The clinical condition on arrival in transported newborn infants after suspected perinatal asphyxia can be difficult to evaluate, not least after administration of anti‐seizure medications during transport. Although the birth history can explain an infant's condition after birth, indicators of perinatal asphyxia need to be verified by more precise clinical evaluation of the infant, including Apgar scores and the presence of early metabolic acidosis.

The present study demonstrated that measurement of LDH on arrival was useful for assessing infants in this study population, as also shown in previous studies. A high LDH together with early depressed electrocortical brain activity, here demonstrated by a depressed aEEG background pattern, was highly predictive of outcome in this population of high‐risk infants.

## Author Contributions

**Hang T. T. Tran:** conceptualization, investigation, methodology, writing – review and editing, formal analysis, project administration, data curation. **Tobias Alfvén:** conceptualization, investigation, writing – original draft, funding acquisition, methodology, validation, supervision. **Ha T. Le:** supervision, project administration, validation, conceptualization. **Dien M. Tran:** supervision, resources, project administration. **Linus Olson:** software, formal analysis, supervision, writing – review and editing, methodology, investigation. **Lena Hellstrom‐Westas:** conceptualization, writing – original draft, writing – review and editing, formal analysis.

## Funding

This study received no specific external funding. The original randomized trial was supported by institutional resources at Vietnam National Children's Hospital and collaborating academic institutions.

## Conflicts of Interest

The authors declare no conflicts of interest.

## Data Availability

The data that support the findings of this study are available on request from the corresponding author. The data are not publicly available due to privacy or ethical restrictions.

## References

[1] A. C. Lee , N. Kozuki , H. Blencowe , et al., “Intrapartum‐ Related Neonatal Encephalopathy Incidence and Impairment at Regional and Global Levels for 2010 with Trends From 1990,” Pediatric Research 74, no. 1 (2013): 50–72. 24366463 10.1038/pr.2013.206 PMC3873711

[2] D. Azzopardi , P. Brocklehurst , D. Edwards , et al., “The TOBY Study. Whole Body Hypothermia for the Treatment of Perinatal Asphyxial Encephalopathy: A Randomised Controlled Trial,” BMC Pediatrics 8 (2008): 17. 18447921 10.1186/1471-2431-8-17 PMC2409316

[3] S. Shankaran , A. Pappas , A. R. Laptook , et al., “Outcomes of Safety and Effectiveness in a Multicenter Randomized, Controlled Trial of Whole‐ Body Hypothermia for Neonatal Hypoxic‐Ischemic Encephalopathy,” Pediatrics 122, no. 4 (2008): e791–e798. 18829776 10.1542/peds.2008-0456 PMC2819143

[4] S. Thayyil , S. Pant , P. Montaldo , et al., “Hypothermia for Moderate or Severe Neonatal Encephalopathy in Low‐Income and Middle‐Income Countries (HELIX): A Randomised Controlled Trial in India, Sri Lanka, and Bangladesh,” Lancet Global Health. 9, no. 9 (2021): e1273–e1285. 34358491 10.1016/S2214-109X(21)00264-3 PMC8371331

[5] H. T. T. Tran , H. T. T. Le , H. T. P. Tran , et al., “Hypothermic Treatment for Neonatal Asphyxia in Low‐Resource Settings Using Phase‐Changing Material‐An Easy to Use and Low‐Cost Method,” Acta Paediatrica 110, no. 1 (2021): 85–93. 32347576 10.1111/apa.15331

[6] H. T. T. Tran , D. M. Tran , H. T. Le , L. Hellström‐Westas , T. Alfven , and L. Olson , “Cooling During Transportation of Newborns with Hypoxic Ischemic Encephalopathy Using Phase Change Material Mattresses in Low‐Resource Settings: A Randomized Controlled Trial in Hanoi,” Vietnam. BMC Pediatr 24 (2024): 509, 10.1186/s12887-024-04987-6. 39118070 PMC11308214

[7] A. I. El Shahed , H. M. Branson , A. Chacko , et al., “Predictive Model of Neurodevelopmental Outcome in Neonatal Hypoxic Ischemic Encephalopathy,” Early Human Development 201 (2025): 106189, 10.1016/j.earlhumdev.2024.106189. 39787883

[8] L. Hellström‐Westas , I. Rosén , and N. W. Svenningsen , “Predictive Value of Early Continuous Amplitude Integrated EEG Recordings on Outcome After Severe Birth Asphyxia in Full Term Infants,” Archives of Disease in Childhood. Fetal and Neonatal Edition 72, no. 1 (1995): F34–F38. 7743282 10.1136/fn.72.1.f34 PMC2528413

[9] S. Reddy , S. Dutta , and A. Narang , “Evaluation of Lactate Dehydrogenase, Creatine Kinase and Hepatic Enzymes for the Retrospective Diagnosis of Perinatal Asphyxia Among Sick Neonates,” Indian Pediatrics 45, no. 2 (2008): 144–147. 18310795

[10] M. Karlsson , E. Wiberg‐Itzel , E. Chakkarapani , M. Blennow , B. Winbladh , and M. Thoresen , “Lactate Dehydrogenase Predicts Hypoxic Ischemic Encephalopathy in Newborn Infants: A Preliminary Study,” Acta Paediatrica 99, no. 8 (2010): 1139–1144. 20236255 10.1111/j.1651-2227.2010.01802.x

[11] H. B. Sarnat and M. S. Sarnat , “Neonatal Encephalopathy Following Fetal Distress. A Clinical and Electroencephalographic Study,” Archives of Neurology 33, no. 10 (1976): 696–705. 987769 10.1001/archneur.1976.00500100030012

[12] H. T. T. Tran , H. T. Le , D. M. Tran , et al., “Therapeutic Hypothermia after Perinatal Asphyxia in Vietnam: Medium‐Term Outcomes at 18 Months ‐ a Prospective Cohort Study,” BMJ Paediatrics Open 8, no. 1 (2024): e002208. 38388007 10.1136/bmjpo-2023-002208 PMC10882320

[13] L. Hellström‐Westas , I. Rosén , L. S. Vries , and G. Greisen , “Amplitude‐Integrated EEG Classification and Interpretation in Preterm and Term Infants,” NeoReviews 7 (2006): 76–87.

[14] T. N. Tsuchida , C. J. Wusthoff , R. A. Shellhaas , et al., “American Clinical Neurophysiology Society Standardized EEG Terminology and Categorization for the Description of Continuous EEG Monitoring in Neonates: Report of the American Clinical Neurophysiology Society Critical Care Monitoring Committee,” Journal of Clinical Neurophysiology 30, no. 2 (2013): 161–173. 23545767 10.1097/WNP.0b013e3182872b24

[15] S. K. Yum , C. J. Moon , Y. A. Youn , and I. K. Sung , “Changes in Lactate Dehydrogenase Are Associated with Central Gray Matter Lesions in Newborns with Hypoxic–Ischemic Encephalopathy,” Journal of Maternal‐Fetal & Neonatal Medicine 30, no. 10 (2017): 1177–1181, 10.1080/14767058.2016.1205022. 27363261

[16] S. Ouwehand , L. C. A. Smidt , J. Dudink , et al., “Predictors of Outcomes in Hypoxic–Ischemic Encephalopathy Following Hypothermia: A Meta‐Analysis,” Neonatology 117, no. 4 (2020): 411–427, 10.1159/00050551. 32235122

[17] R. Del Río , C. Ochoa , A. Alarcon , J. Arnáez , D. Blanco , and A. García‐Alix , “Amplitude Integrated Electroencephalogram as a Prognostic Tool in Neonates with Hypoxic‐Ischemic Encephalopathy: A Systematic Review,” PLoS One 11, no. 11 (2016): e0165744. 27802300 10.1371/journal.pone.0165744 PMC5089691

[18] S. K. Yum , C. J. Moon , Y. A. Youn , and I. K. Sung , “Changes in Lactate Dehydrogenase Are Associated with Central Gray Matter Lesions in Newborns with Hypoxic‐Ischemic Encephalopathy,” Journal of Maternal‐Fetal & Neonatal Medicine 30, no. 10 (2017): 1177–1181. 27363261 10.1080/14767058.2016.1208745

[19] A. Mehta , D. Chawla , J. Kaur , V. Mahajan , and V. Guglani , “Salivary Lactate Dehydrogenase Levels Can Provide Early Diagnosis of Hypoxic‐Ischemic Encephalopathy in Neonates with Birth Asphyxia,” Acta Paediatrica 104, no. 6 (2015): e236–e240. 25656073 10.1111/apa.12964

[20] M. Thoresen , X. Liu , S. Jary , et al., “Lactate Dehydrogenase in Hypothermia‐Treated Newborn Infants with Hypoxic‐Ischemic Encephalopathy,” Acta Paediatrica 101, no. 10 (2012): 1038–1044. 22775455 10.1111/j.1651-2227.2012.02778.x

[21] M. Thoresen , “Patient Selection and Prognostication with Hypothermia Treatment,” Seminars in Fetal & Neonatal Medicine 15, no. 5 (2010): 247–252. 20580626 10.1016/j.siny.2010.05.008

